# T-Cell-Driven Inflammation as a Mediator of the Gut-Brain Axis Involved in Parkinson's Disease

**DOI:** 10.3389/fimmu.2019.00239

**Published:** 2019-02-15

**Authors:** Javier Campos-Acuña, Daniela Elgueta, Rodrigo Pacheco

**Affiliations:** ^1^Laboratorio de Neuroinmunología, Fundación Ciencia and Vida, Ñuñoa, Santiago, Chile; ^2^Departamento de Ciencias Biológicas, Facultad de Ciencias de la Vida, Universidad Andres Bello, Santiago, Chile

**Keywords:** CD4^+^ T-cell mediated immunity, neo-antigens, gut microbiota, gut-brain axis, Parkinson's disease, neuroinflammation

## Abstract

Parkinson's disease (PD) is a neurodegenerative disorder affecting mainly the dopaminergic neurons of the nigrostriatal pathway, a neuronal circuit involved in the control of movements, thereby the main manifestations correspond to motor impairments. The major molecular hallmark of this disease corresponds to the presence of pathological protein inclusions called Lewy bodies in the midbrain of patients, which have been extensively associated with neurotoxic effects. Importantly, different research groups have demonstrated that CD4^+^ T-cells infiltrate into the substantia nigra of PD patients and animal models. Moreover, several studies have consistently demonstrated that T-cell deficiency results in a strong attenuation of dopaminergic neurodegeneration in animal models of PD, thus indicating a key role of adaptive immunity in the neurodegenerative process. Recent evidence has shown that CD4^+^ T-cell response involved in PD patients is directed to oxidised forms of α-synuclein, one of the main constituents of Lewy bodies. On the other hand, most PD patients present a number of non-motor manifestations. Among non-motor manifestations, gastrointestinal dysfunctions result especially important as potential early biomarkers of PD, since they are ubiquitously found among confirmed patients and occur much earlier than motor symptoms. These gastrointestinal dysfunctions include constipation and inflammation of the gut mucosa and the most distinctive pathologic features associated are the loss of neurons of the enteric nervous system and the generation of Lewy bodies in the gut. Moreover, emerging evidence has recently shown a pivotal role of gut microbiota in triggering the development of PD in genetically predisposed individuals. Of note, PD has been positively correlated with inflammatory bowel diseases, a group of disorders involving a T-cell driven inflammation of gut mucosa, which is strongly dependent in the composition of gut microbiota. Here we raised the hypothesis that T-cell driven inflammation, which mediates dopaminergic neurodegeneration in PD, is triggered in the gut mucosa. Accordingly, we discuss how structural components of commensal bacteria or how different mediators produced by gut-microbiota, including short-chain fatty acids and dopamine, may affect the behaviour of T-cells, triggering the development of T-cell responses against Lewy bodies, initially confined to the gut mucosa but later extended to the brain.

## Introduction

Parkinson's disease (PD) is the second most common neurodegenerative disorder in the world, which involves the progressive death of dopaminergic neurons in the nigrostriatal pathway, a midbrain circuit responsible for the control of voluntary movements. Accordingly, this disorder is characterised by motor symptoms such as bradykinesia, tremor, and postural abnormalities (1). In addition to the motor symptoms, one of the main hallmarks of PD is the presence of protein aggregates in the brain of patients, which are known as Lewy bodies. Importantly, α-synuclein, a central molecular player involved in the physiopathology of PD, has been found to be the main component of Lewy bodies (2). The process of α-synuclein aggregation to form Lewy bodies and the generation of intermediaries oligomers have been associated with the neurotoxic mechanisms involved in PD (3). Of note, one of the main causes of Lewy bodies generation is the oxidative stress, which promotes the covalent modifications of α-synuclein (i.e., by nitration) which strongly favour aggregation (4). In this regard, it has been proposed that aggregation represents an aberrant folding that competes with the proper healthy folding of α-synuclein favoured by high chaperone activity and low oxidative stress. Thereby, an overload of α-synuclein, decreased redox capability or reduced chaperone activity would make neurons more prone to α-synuclein aggregation (5, 6).

Intriguingly, most PD patients present a number of non-motor manifestations such as insomnia, loss of smell, anxiety, depression, apathy, and gastrointestinal dysfunctions, which commonly precede motor symptoms by several years (7, 8). Among non-motor manifestations, gastrointestinal dysfunctions result especially important as potential early biomarkers of PD, since they are ubiquitously found among confirmed patients and occurs much earlier than motor symptoms (9). In this regard, several lines of evidence have suggested a causal relationship between the gut and the brain in PD (10–13). The hypothesis of the involvement of a gut-brain axis was initially proposed by Braak and collaborators in which they suggested that environmental pathogens would be able to cross the intestinal epithelium and to induce misfolding and aggregation of α-synuclein in specific neurons of the enteric nervous system and subsequently, aggregated α-synuclein would propagate to the brain by migrating through the vagus nerve (14). Supporting this idea, enteric neurons have been found to be able to secrete α-synuclein (15). Moreover, experimental evidence obtained in rodents has shown that aggregated α-synuclein administered in the gut might promote aggregation of endogenous α-synuclein and the propagation of these aggregates through the vagus nerve contributing to the accumulation of aggregated α-synuclein in the brain (16). Notably, aggregated α-synuclein reaching the central nervous system might further spread transneuronally to different areas of the brain (17). It has also been observed the loss of neurons of the myenteric and submucosal plexi at early stages of PD, which is associated with apparition of Lewy bodies in the dorsal motor nucleus of the vagus nerve and decreased gastrointestinal motility (18–20). Furthermore, according to the Braak's hypothesis, it has been found that vagotomy in humans results in a reduced risk of PD (21). Importantly, studies performed in rodent models have shown that direct lesion of the nigrostriatal pathway results in altered colonic physiology and, on the other hand, colon inflammation triggers disturbance of nigrostriatal homeostasis, thus indicating a bidirectional communication between central dopaminergic neurons and the enteric nervous system (12).

Increased gut permeability, which corresponds to one of the main triggers of gut inflammation, has been also found in early diagnosed PD patients, a process that correlates with enhanced accumulation of α-synuclein in the gut mucosa (22). Accordingly, it has been described that PD patients display high levels of pro-inflammatory cytokine expression (TNF-α, IFN-γ, and IL-6) and glial activation markers (GFAP and Sox-10) in the ascending colon, thereby indicating an association between PD and colonic inflammation (23). In this regard, a recent study developed with more than 23 million individuals has shown that inflammatory bowel diseases (IBD) represent a risk factor to develop PD (24). Moreover, subsequent studies have shown a significant reduction in the risk to develop PD in those IBD patients that received early treatments with anti-inflammatory therapies such as anti-TNF-α or underwent surgery where tissue with high concentrations of α-synuclein aggregates was removed, thus limiting Lewy bodies spreading to the brain (25, 26).

Bacterial products also play a key role in the development of inflammation in the gut and the brain. Increased gut permeability promotes the leakage of bacteria and their products into the blood circulation leading to the maturation of antigen-presenting cells and the consequent stimulation of inflammatory pathways, thus triggering oxidative stress and favouring the accumulation of aggregated α-synuclein in the enteric nervous system (22, 27). Moreover, systemic inflammation triggered by bacterial products in the blood stream may induce a strong production of pro-inflammatory cytokines by cells from the innate and the adaptive immune system, which can spread through the blood, favour the permeabilization of the blood-brain-barrier and reach the brain. The subsequent stimulation of glial cells by pro-inflammatory cytokines coming from the periphery can trigger neuroinflammation and consequent neuronal death, such as the case of neurodegeneration induced by intraperitoneal administration of lipopolysaccharide (LPS) (28). Furthermore, it is important to consider that several studies performed in recent years have shown a strong influence of intestinal microbiota in the control of gut inflammation (29–32). Of note, emerging evidence has shown that gut microbiota may control inflammation in two ways: (1). By producing a milieu of mediators that exert direct effects stimulating their receptors in eukaryotic cells of the host, such as short chain fatty acids (SCFAs), neurotransmitters and other metabolites (29, 33); and (2). By providing structures with molecular mimicry with self-antigens, which trigger activation of T-cells with autoreactive potential (34, 35). Importantly, a number of studies have shown relevant association of intestinal microbiota composition with the development of PD (10, 11, 13). However, it is still not clear whether these changes in the composition of the gut microbiota involve the generation of a milieu of microbiota-derived mediators that promote inflammatory behaviour in T-cells, the presence of molecular components with mimicry with self-antigens (i.e., Lewy bodies) or both.

Taken together these findings we propose here the hypothesis that CD4^+^ T-cell response to Lewy bodies derived antigens is involved in the connection between inflammation in the gut and inflammation in the brain in the context of PD. Accordingly, in this review we analyse how commensal microbiota and its metabolites in the gut may participate triggering α-synuclein aggregation, the main source of antigens driving T-cell-mediated inflammation in PD. We also discuss the evidence indicating that microbiota-derived metabolites may affect the inflammatory and the suppressive function of T-cells, thus suggesting that the precise composition of the microbial consortium in the gut might break the tolerance to self-antigens, triggering T-cell-driven autoimmunity. Finally, we analyse the mechanisms of how dopamine and SCFAs, two key mediators strongly affected by the composition of gut microbiota, may affect T-cell behaviour, and how the alteration in the levels of these mediators might be involved in the development of T-cell mediated autoimmunity associated to PD.

## T-cell Driven Inflammation Plays a Fundamental Role in the Physiopathology of IBD and PD

Evidence in human and animal models has shown the generation of oxidised forms of α-synuclein, especially nitrated α-synuclein, in the substantia nigra of individuals with PD (36–38), which constitutes a major component of Lewy bodies. Of note, the nitration of α-synuclein, which is a consequence of the oxidative stress, results in the generation of neo-antigens (4). Furthermore, studies in mice and recently in humans, have shown that oxidised α-synuclein constitutes a major antigen for the T-cell-mediated immune response involved in PD (37, 39–41). In this regard, it has been shown that nitrated α-synuclein generated in the substantia nigra is captured and presented by antigen-presenting-cells (APCs) in cervical lymph nodes to naive CD4^+^ T-cells with specificity to this neo-antigen. Once activated, CD4^+^ T-cells acquire inflammatory phenotypes, such as T-helper-1 (Th1) and Th17, they infiltrate the substantia nigra where microglial cells act as local APCs presenting peptide-antigens derived from nitrated α-synuclein on class II MHC, thus re-stimulating T-cells (37, 42–44). Restimulated CD4^+^ T-cells produce high local levels of IFN-γ and TNF-α, thus promoting further inflammatory activation of microglial cells (M1 microglia) (45–48). Activated M1 microglia produces high levels of glutamate, TNF-α and reactive oxygen and nitrogen species (ROS/RNS), which in turn induce neuronal death and further generation of oxidised and nitrated proteins, including nitrated α-synuclein (4). Initial microglial activation makes blood-brain barrier (BBB) permissive for leukocyte entrance, and cytokines produce by Th1 and Th17 cells recruit and activate peripheral monocytes/macrophages and neutrophils which produce further neuronal damage (48). Thus, this mechanism involving the innate and adaptive immune system constitutes a vicious cycle, which results in chronic neuroinflammation and constitutes the engine of the progression of neurodegeneration. Of note, several studies performed with different T-cell deficient mouse strains, including TCR-β-chain deficient mice, severe combined immunodeficiency (SCID) mice and recombination-activating-gen-1 (RAG1) knockout (RAG1KO) mice, have shown that T-cell deficiency results in a complete protection of neurodegeneration in mouse models of PD (37, 46, 49). Furthermore, additional analyses have shown that whereas CD8-deficiency does not affect the extent of dopaminergic neurodegeneration, CD4-deficiency results in a strong attenuation of neurodegeneration in a mouse model of PD induced by MPTP (49), thus suggesting that inflammatory CD4^+^ T-cell response plays a fundamental role promoting neurodegeneration of the nigrostriatal pathway.

Similar to the case of PD, several studies performed with inflammatory colitis mouse models and with samples obtained from patients with Ulcerative Colitis (UC) and with Crohn's disease (CD) have consistently indicated that gut inflammation in IBD is driven mainly by the inflammatory effector CD4^+^ T-cell subsets Th1 and Th17 (50, 51). In addition, regulatory CD4^+^ T-cells (Tregs), a suppressive subset of lymphocytes, seem to play a crucial role in maintaining intestinal homeostasis. These cells can suppress inflammation induced by effector T-cells (Th1 and Th17) in a mouse model of chronic inflammatory colitis induced by T-cell transfer into lymphopenic mice (52); and one of the main suppressive mechanisms relies on IL-10 secretion by these cells. In humans, Tregs are increased in the inflamed lamina propria of CD and UC patients compared to uninflamed mucosa and mucosa from healthy controls, and after isolation they retain their ability to suppress effector T-cell response *in vitro*, suggesting that Tregs function could be attenuated just *in situ* by mediators produced by the inflamed gut mucosa (53). Similar to the beneficial role of Tregs in mucosal immunity, the Th22 subset of CD4+ T-cells has been shown to promote homeostasis. In this regard, IL-22 produced by these cells induces the expression of tight junction proteins (i.e., claudin 1 and ZO-1) in epithelial cells, thus increasing the integrity of the mucosal epithelial barrier and protecting it from inflammation (54). Accordingly, it has been shown that the administration of anti-TNF-α therapy (infliximab) in CD patients, which ameliorates gut inflammation, upregulates IL-22 production contributing to intestinal epithelial barrier repair (54). Regarding the antigens recognised by the adaptive immune system in IBD, several autoantigens and microbiota-derived antigens have been described in both CD and UC (55, 56). In the case of animal models of inflammatory colitis induced by different approaches, the main antigens recognised by adaptive immune system have been shown to correspond to microbiota-derived antigens. For instance, colitis induced by administration of chemicals such as dextran sodium sulfate or 2,4,6-trinitrobenzene sulfonic acid involve the disruption of epithelial layer of gut mucosa, resulting in an acute inflammatory response against microbiota-derived antigens (57, 58). In the case of genetic deficiency of IL-10, the inflammatory response in the gut is caused by the lack of the main suppressive mechanism used by gut Tregs to maintain mucosal homeostasis (59). The model of inflammatory colitis induced by T-cell transfer involves the administration of naive CD4^+^ T-cells into lymphopenic recipient mice (60). In these conditions, most naive CD4^+^ T-cells become activated in the gut-associated secondary lymphoid organs by recognising microbiota-derived antigens in the absence of Tregs. Activated CD4^+^ T-cells differentiate in Th1 and Th17 cells, infiltrate the colonic lamina propria and release IFN-γ, IL-17, and other inflammatory mediators that recruit and stimulate neutrophils and macrophages, thus inducing chronic inflammation in gut mucosa (60). Considering the significant association between PD and IBD, it is likely that Lewy bodies derived antigens might be important targets for the adaptive immune system in IBD as well. According to this notion, it has been hypothesised that upon disruption of the epithelial layer of gut mucosa some microorganisms might induce inflammation, thus promoting oxidative stress and the consequent aggregation of α-synuclein produced by neurons of the enteric nervous system (14, 15). Another possible mechanism to explain how microenvironmental microorganisms might trigger an adaptive immune response against Lewy bodies is by molecular mimicry. In this regard, it has been shown that herpes simplex virus 1 (HSV1) derived antigens trigger the activation of homologous T-cells and B-cells that recognise α-synuclein derived antigens (61, 62). Furthermore, a study that analysed the seropositivity of PD patients and healthy controls to common infectious agents showed that the infection burden of HSV1 and some other pathogens is associated with PD (63). Thus, HSV1 infection might represent an environmental factor triggering PD and/or IBD in genetically susceptible individuals with proper MHC molecules able to present HSV1-derived peptides with molecular mimicry with α-synuclein-derived peptides. We further develop the discussion about potential involvement of molecular mimicry in the section Involvement of Gut-Microbiota in Autoimmunity. Taken together, the evidence indicates that T-cell driven inflammation represents a central process in both, PD and IBD, and suggests that Lewy bodies derived antigens might be important targets leading this T-cell mediated immunity.

## Lewy Bodies as Triggers of T-cell Mediated Immunity

As stated above, pathological inclusions of α-synuclein appear in early stages of PD, forming Lewy bodies in cells of the enteric nervous system (9). It has been proposed that initial α-synuclein aggregation and consequent Lewy bodies generation would take place in neurons of sites exposed to hostile environmental factors such as the olfactory bulb and gastrointestinal tract (64). Afterward, these α-synuclein inclusions would be transported from the peripheral nervous system to the brain by axonal retrograde movements, a hypothesis supported by experimental data obtained in rodents (16). In this regard, it has been shown that after the injection of human α-synuclein in the gut of rats, this protein is transported through the vagus nerve, reaching the brainstem (16). Furthermore, a number of mouse models of PD that recapitulate the accumulation of aggregated α-synuclein show similar spatiotemporal patterns of Lewy bodies formation as those observed in PD patients, beginning with Lewy bodies generation in the gut several months before the manifestation of motor symptoms (10, 65, 66). The observation that Lewy bodies appearance takes place early in the gut mucosa, even before Lewy bodies formation in the brain suggests that the generation of Lewy bodies would be triggered by environmental factors present in the gut, such as the gut microbiota. Supporting this idea, it has been shown that transgenic mice overexpressing human α-synuclein generate aggregates of α-synuclein in the gut and the brain and develop several Parkinsonian symptoms when housed in specific pathogen free conditions. Nevertheless, these mice display a strong attenuation in α-synuclein aggregation and motor impairment when microbiota is depleted by treatment with broad-spectrum antibiotics or when they are bred in germ free conditions (10). Another study supporting the idea that Lewy bodies aggregation is induced by environmental factors in genetically susceptible individuals has been performed in transgenic mice expressing a A53T mutant form of α-synuclein treated with Paraquat. This drug is a pesticide that is mainly ingested through airways and exerts the inhibition of mitochondrial respiration promoting oxidative stress (67). Oral administration of paraquat triggers expression of aggregated α-synuclein in the olfactory bulb and the enteric nervous system of transgenic mice earlier than in the brain and manifestation of motor impairment (68).

Importantly, several lines of evidence have shown that aggregated α-synuclein might act as a neo-antigen able to trigger adaptive immunity. Accordingly, Lewy bodies have been shown to stimulate Toll-like receptors 2 (TLR2) and TLR4 in local glial cells as well as in infiltrating APCs (i.e., monocyte/macrophages and dendritic cells), thus inducing an initial inflammation in the microenvironment where aggregated α-synuclein is generated (4). Of note, TLRs signalling induces NF-κB activation, which triggers the acquisition of inflammatory phenotypes by glial cells and the acquisition of immunogenic features by dendritic cells involving high expression of class II MHC and strong co-stimulation (47). In line with the idea that PD is initiated in the gut, it has been shown that oral administration of paraquat in transgenic A53T mice triggers an increased expression of Glial fibrillary acidic protein (GFAP; a classic activation marker of astrocytes) in glial cells of the enteric nervous system before neuropathology manifestation in the brain (68). Moreover, a study performed with endoscopic biopsies of children with gut inflammation shows a significant correlation between the levels of α-synuclein accumulation in neurites of the enteric nervous system and the degree of inflammation of intestinal wall (69). Of note, an equivalent local inflammatory reaction is observed in the substantia nigra upon direct delivery of aggregated α-synuclein. In this regard, the stereotaxic injection of α-synuclein fibrils in the substantia nigra of rats induces an increased expression of class II MHC (an activation marker of glial cells) in local microglial cells of the nigrostriatal pathway as well as the recruitment of peripheral APCs and lymphocytes (70). Taken together these findings indicate that gut microbiota plays an important role as an environmental factor inducing local α-synuclein aggregation, which in turn triggers the stimulation of TLRs in innate immune cells, thus promoting an initial inflammation in the gut mucosa.

**Figure 1:**
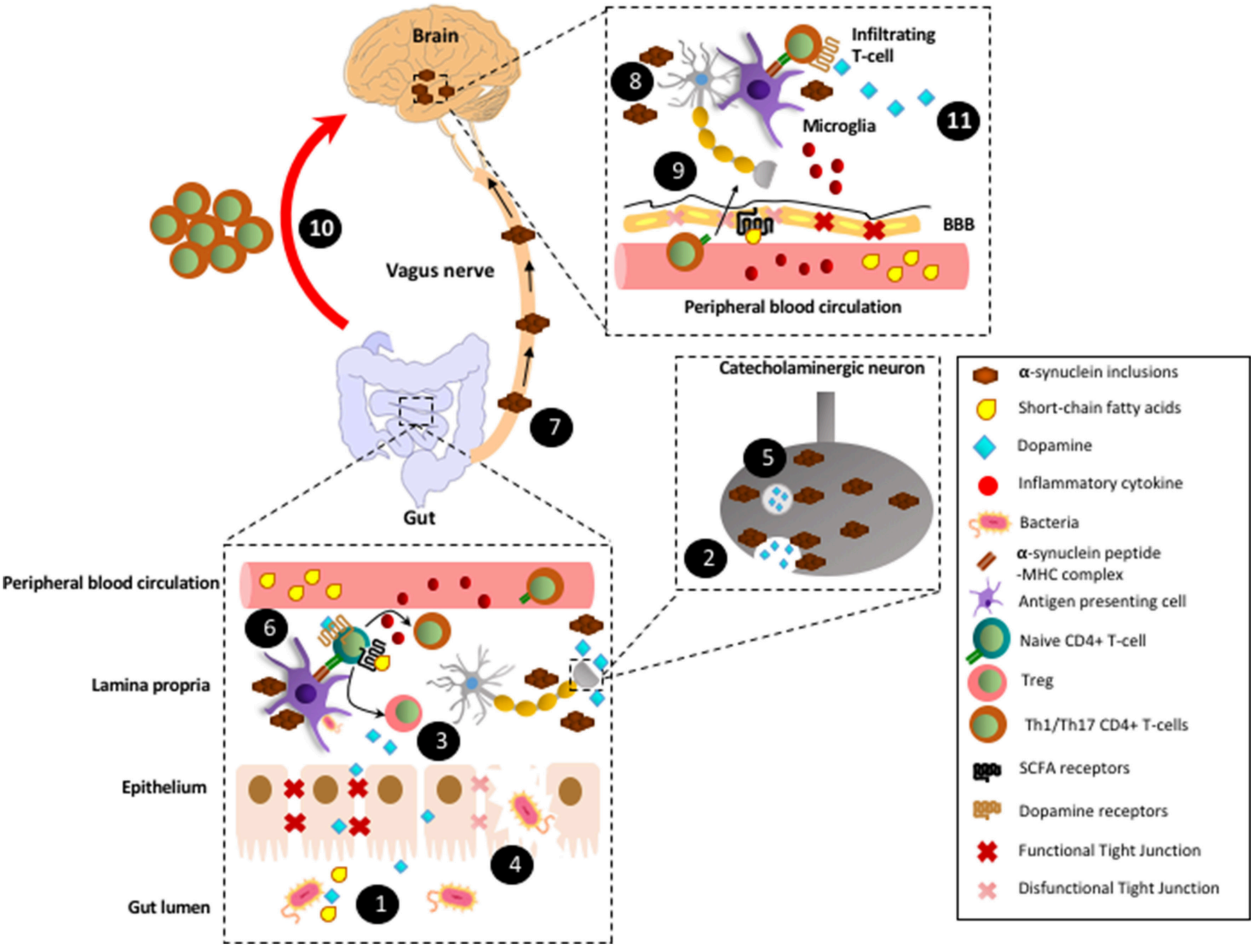
Proposed model by which CD4^+^ T-cell response involved in Parkinson's disease are triggered in the gut mucosa. (1) In healthy conditions, gut microbiota produces SCFAs and high levels of dopamine. (2) In addition to the gut microbiota, catecholaminergic neurons of the enteric nervous system also contribute to the secretion of high dopamine levels into the gut mucosa and lumen. (3) SCFAs and dopamine stimulate GPR41, GPR43, and GPR109A and low-affinity dopamine receptors (i.e., DRD2), respectively in CD4^+^ T-cells, favouring their differentiation into Tregs and their suppressive activity, thereby promoting tolerance to food-derived and microbiota-derived antigens. (4) Under some circumstances, such as dysbiosis, some tight junctions components become down-regulated and thereby epithelial layer of gut mucosa might be disrupted. Consequently, some strains of gut microbiota trigger an initial inflammation mediated by innate immune cells, which promote local oxidative stress with the covalent modification of self-proteins. (5) The oxidative environment promotes the generation of α-synuclein inclusions, which impair vesicular secretion by neurons of the enteric nervous system and thereby reduction in dopamine levels. (6) In addition, α-synuclein inclusions are captured by mucosal APCs and presented to naïve CD4^+^ T-cells specific for Lewy bodies derived antigens. Moreover, α-synuclein inclusions stimulates TLRs in macrophages and dendritic cells, triggering thus inflammation, oxidative stress and thereby further generation of α-synuclein inclusions, which constitutes a vicious cycle of chronic inflammation and generation of Lewy bodies. (7) According to Braak's hypothesis, after a long period of time (years) with chronic inflammation, Lewy bodies generated in the enteric nervous system would be transported by retrograde movement through vagus nerve until reaching the brain stem. (8) Lewy bodies in the brain would stimulate TLRs in microglial cells inducing the production of inflammatory cytokines and thus favouring the permeabilization of the BBB. (9) Inflammatory cytokines coming from peripheral blood circulation would also contribute to BBB permeabilization. In addition, a reduction in SCFAs (i.e., induced by a dysbiosis in gut microbiota) might alter GPR41-signalling in the BBB, thus promoting disassembling of tight junctions and further permeabilization of the BBB. (10) Inflammatory CD4^+^ T-cells (Th1 and Th17) generated years ago in response to Lewy bodies in the gut mucosa would migrate through the blood and infiltrate the brain (red arrow; this is the main hypothesis raised here). (11) Microglial cells would capture Lewy bodies and subsequently they would present Lewy bodies-derived antigens to Th1 and Th17 infiltrating the brain. Thus, microglial cells would restimulate Lewy body-specific CD4^+^ T-cells promoting further neuroinflammation and neurodegeneration of the dopaminergic neurons of the nigrostriatal pathway.

Importantly, one of the main consequences of TLR stimulation in APCs is an increased expression of class II MHC molecules on the cell surface (4, 70). Thus, mucosal dendritic cells present in those zones of the gut where α-synuclein aggregation is initiated would receive TLR-stimulation and concomitantly would capture Lewy bodies, process them intracellularly to yield small peptides, and subsequently would present Lewy bodies derived peptides on class II MHC. TLR-stimulation also induces a fast migration of dendritic cells into the draining lymph nodes (MLN in the case of colonic mucosa) and thus, Lewy bodies derived antigens would be presented on class II MHC to naïve CD4^+^ T-cells, which normally keep patrolling though these lymphoid tissues (71). In this way, when naïve CD4^+^ T-cells bearing TCR specific for the recognition of Lewy bodies-derived antigens appear and recognise their antigen presented by dendritic cells, they would become activated, proliferate, and then would migrate to the site where inflammation has been initiated, in this case the gut mucosa (72). Supporting this notion, it has recently been described the presence of inflammatory CD4^+^ T-cells with specificity by different Lewy bodies derived antigens in PD patients (41). Since dendritic cells would acquire an immunogenic phenotype upon TLR-stimulation, including the production of inflammatory cytokines and high expression of class II MHC and co-stimulation, Lewy bodies derived antigens presentation should induce inflammatory effector phenotypes in antigen-specific CD4^+^ T-cells. Accordingly, recent studies analysing the peripheral immune system of PD patients have shown a biased Th1 (73) and Th17 immunity (74). Th1 and Th17 have been extensively involved in autoimmunity, where they infiltrate the target tissue and promote: (i) The recruitment of monocytes and neutrophils, respectively and; (ii) The microbicide and oxidative activity of local macrophages and infiltrated monocytes and neutrophils, thus favouring local inflammation and tissue damage (75). Therefore, the ROS and RNS induced in local phagocytes by Th1 and Th17 immunity would promote further aggregation of α-synuclein in the neurons of the enteric nervous system. This mechanism would represent a vicious cycle, which results in chronic inflammation, further generation of Lewy bodies in the gut, and the subsequent spreading of Lewy bodies to the brainstem as suggested before (14). Once Lewy bodies reach the brain, they might stimulate TLR signalling in microglial cells, thus favouring the permeabilization of the BBB and the subsequent recruitment of Lewy bodies-specific Th1 and Th17 cells, promoting brain damage (4). Considering all of these factors, we propose the following model: the initial aggregation of α-synuclein in the gut mucosa would trigger Th1 and Th17 immunity and further generation of Lewy bodies that would migrate to the brain in later stages. In this way, T-cell mediated inflammation would represent the engine of tissue damage, first in the gut and later in the brain (Figure 1).

According to the coordinate production of autoantibodies commonly associated to T-cells mediated autoimmunity and to the autoimmune nature of PD, autoreactive B-cells have also been involved in this pathology (4). Accordingly, the presence of autoantibodies in the serum and infiltrated within the brain parenchyma has been described in PD (76, 77). Furthermore, analyses performed in necropsies of PD patients have shown that immunoreactivity associated to autoantibodies is located specifically in dopaminergic neurons and Lewy bodies (77). Moreover, the high degree of FcγRs detected on microglia and mononuclear cells infiltrating the substantia nigra of PD patients (77) suggests that the infiltration of these autoantibodies into the central nervous system may strongly contribute to neuroinflammation and neurodegeneration. Despite there are available evidence involving autoantibodies directed to Lewy bodies in the central nervous system of PD patients, there are not studies addressing the presence of autoantibodies to Lewy bodies in gut inflammation.

## Gut Microbiota as a Master Regulator of T-cell Mediated Immunity

Intestinal microbiota is in close contact with the gut epithelial barrier, which isolates and separates the intestinal lumen from the rest of the organism. Interestingly, hosts organisms have evolved together with commensal bacteria to establish a symbiotic relationship by mean of synthesising and responding to several common mediators (78). In this regard, mammals have evolved to take advantage of the presence of commensal microbiota in the gastrointestinal tract far beyond the simple degradation of nutrients by some types of bacteria, as mediators synthesised by gut microbiota, including neurotransmitters, metabolites, and fatty acids can strongly affect host metabolism, neural circuits, hormone secretion, behaviour, and the immune response (78, 79). Accordingly, pathologic alterations in the composition of gut microbiota (dysbiosis) have been strongly involved in the development of cancer as well as neuropsychiatric, metabolic, autoimmune and neurodegenerative disorders (80–82). Three mechanisms have been proposed to explain the influence that gut microbiota exerts in the host physiology: (i) The first one involves the secretion of neurotransmitters, neuropeptides and metabolites that might directly stimulate their receptors in neurons of the enteric nervous system, thus triggering and/or modulating neural signals that affect directly the gut physiology or migrate through vagal transmission to the central nervous system affecting behaviour (78). (ii) The second mechanism proposed involves metabolites and hormones produced by microbiota in the intestinal tract that might diffuse through the gut wall, entering into the portal circulation and then exert their effects far away by stimulating their receptors expressed in other organs such as adrenal gland, liver, or others (78). (iii) The third mechanism proposed involves the stimulation of receptors expressed in immune cells by mediators produced by gut microbiota, such as short-chain fatty acids (SCFAs), neurotransmitters and other metabolites (29, 83), thus shaping the immune response (see sections Dopaminergic Regulation of T-cell Mediated Immunity and Short-Chain Fatty Acids as Regulators of T-cell Mediated Immunity).

Mucosal immunity involves a tight equilibrium between inflammatory responses against orally administered dangerous foreign antigens and the generation of tolerance to food-derived and commensal microbiota-derived antigens. Regarding the role of gut-microbiota in the immune system, several studies have extensively shown a key role of intestinal segmented filamentous bacteria (SFB) in the induction of Th17 cells in the gut mucosa (84, 85). Interestingly, it has been shown that signals triggered by SFB to induce Th17 differentiation does not depend on receptors of the innate immune system (i.e., TLRs), but strongly depends on the adhesion of SFB to the intestinal epithelial cells (86). In homeostatic conditions this inflammatory subset of CD4^+^ T-cells, controls the invasion of gut mucosa by several pathogenic bacterial species by inducing the secretion of IgA by plasma cells into the colonic lumen. Besides mediating immunity against pathogenic bacteria, the induction of Th17 cells in the gut mucosa has also been associated with the development of autoimmune disorders, such as multiple sclerosis, arthritis and uveitis (34, 87, 88), making them a double-edged sword if not controlled properly. In addition to the control of inflammatory Th17 cells, gut microbiota plays also an important role favouring the activity of immunosuppressive T-cells to promote tolerance to innocuous antigens derived from food and commensal bacteria (29). For instance, it has been shown that the commensal bacterium *Bacteroides fragilis* induces the generation of extra-thymic Foxp3+ Tregs in the intestine, thus favouring a tolerogenic environment in the gut mucosa. It is noteworthy that mono-colonization of germ-free animals with *Bacteroides fragilis* significantly increase the IL-10 production and the suppressive activity of Tregs (exclusively in the Foxp3+ population) in the gut mucosa, an effect mediated by the stimulation of TLR2 in mucosal APCs by polysaccharide-A expressed on *Bacteroides fragilis* (89). Thus, these examples illustrate how individual components of the gut microbiota can exert strong changes in the outcome of mucosal immunity.

Addressing the relevance of commensal bacteria in the development of Parkinson's disease, a recent study was carried out using a transgenic mice over-expressing human α-synuclein (ASO mice) in which microbiota was depleted. ASO mice spontaneously develop neuroinflammation, generation of α-synuclein inclusions in the nigrostriatal pathway and several parkinsonian sympoms after 12 weeks of age, including motor impairment and intestinal dysfunction (90). Strikingly, it was shown that depletion of microbiota, by the treatment of mice with broad-spectrum antibiotics or by breeding them in germ-free conditions, results in a nearly complete abolition of the Parkinsonism manifestation, including the attenuation of α-synuclein inclusions generation, neuroinflammation, and motor and intestinal impairment (10). Moreover, when germ-free ASO mice were repopulated with gut microbiota obtained from PD patients they developed a stronger parkinsonian phenotype than when repopulated with gut microbiota obtained from healthy human individuals (10). Interestingly, a distinctive product from microbiota obtained from PD patients in comparison with microbiota obtained from healthy controls was the production of SCFAs, including butyrate, propionate, and acetate. Notably, treatment of germ-free ASO mice with SCFAs recapitulated the pathogenic effect of gut microbiota triggering the development of parkinsonian phenotype (10). A later study addressed the question of whether there is a particular commensal bacterium responsible for triggering the development of PD. Interestingly, the authors found that *Proteus mirabilis* was particularly increased in the gut microbiota of a number of different PD mouse models, including the MPTP (1-methyl-4-phenyl-1,2,3,6-tetrahydropyridine) model, the MPTP plus probenecid model and the 6-OHDA (6-hydroxydopamine) model (11). The oral administration of *Proteus mirabilis* promotes α-synuclein aggregation in the gut and the brain, favours neuroinflammation and neurodegeneration of dopaminergic neurons of the nigrostriatal pathway and exacerbates the motor impairment (11). The analysis of the molecular mechanism underlying revealed that LPS expressed by *Proteus mirabilis* induced a down-regulation of occludin expression in the gut mucosa, thus disassembling tight junctions in the colon and favouring the disruption of epithelial intestinal layer (11), which triggers an inflammatory process as described in the section Lewy Bodies as Triggers of T-cell Mediated Immunity. According to these results, another study has shown that fecal microbiota transplantation from healthy controls significantly reduces the dysbiosis in PD animals and attenuates the extent of neurodegeneration, neuroinflammation, and motor impairment (13). These findings together indicate that some particular components of the gut microbiota might be the triggers of α-synuclein aggregation and subsequent PD development. However, is important to keep in mind that gut commensal microbiota is composed by more than 1,000 different bacterial species and thereby it is expected that they together should produce complex milieu of mediators that might affect the behaviour of the immune system. In this regard, next sections focus in the analysis of two kind of molecular cues strongly affected by the microbiota composition and that exert key effects on the adaptive immune response: dopamine and SCFAs.

## Dopaminergic Regulation of T-cell Mediated Immunity

Gut mucosa, which plays a critical role in the induction of tolerance to dietary antigens and to commensal microbiota, constitutes a major source of dopamine available for immune cells (91–93). Importantly, dopamine-mediated regulation of immunity in the gut mucosa seems to be critical for maintaining the tolerance to innocuous antigens, as gut dopamine levels are strongly reduced in patients with CD and UC and in animal models of inflammatory colitis (91, 92). Gut dopamine might be produced from different sources, including the intrinsic enteric nervous system, the intestinal epithelial layer (94), some components of the gut microbiota (95), and certain immune cells, including dendritic cells and Tregs (96–98). Nevertheless, the evidence indicates that one of the main sources of dopamine present in the gut mucosa is given by the commensal gut microbiota (93). In this regard, it has been described that most dopamine arrives to the gut mucosa as glucuronide conjugated, which is biologically inactive. Nevertheless, *Clostridium* species present in the gut microbiota express β-glucuronidase activity, which catalyses the production of free dopamine in the gut mucosa (93). In addition, recent studies have shown *in vitro* evidence indicating that some components of gut microbiota, including *Bacillus cereaus, Bacillus mycoides, Bacillus subtilis, Proteus vulgaris, Serratia marcescens, S. aureus, E. coli* K-12, *Morganella morganii, Klebisella pneumonia*, and *Hafnia alvei*, can also produce dopamine (95). Interestingly, similar to the situation observed in IBD, striatal dopamine levels are also significantly reduced in PD (49), a process that can be observed even before the degeneration of dopaminergic neurons of the nigrostriatal pathway (99). Of note, both IBD and PD involve a local inflammation driven by CD4^+^ T-cells (as discussed in section Lewy Bodies as Triggers of T-cell Mediated Immunity), cells that are thereby exposed to these changes in dopamine levels. Dopamine exerts its effects by stimulating DRs, termed DRD1-DRD5; all of them belonging to the superfamily of G-protein coupled receptors. All these receptors have been found in CD4^+^ T-cells from human and mouse origin (100). It is important to consider that each DR displays different affinities for dopamine: DRD3>DRD5>DRD4>DRD2>DRD1 (Ki(nM) = 27, 228, 450, 1,705, 2,340, respectively), thereby their functional relevance depend on dopamine levels (83). Regarding the role of DRs expressed in CD4^+^ T-cells upon inflammation, our recent studies showed that DRD3-deficient naïve CD4^+^ T-cells display impaired Th1 differentiation and reduced expansion of Th17 cells and consequently an attenuated manifestation of inflammatory colitis (101, 102). Taking into account the reduction in intestinal dopamine levels [≈1,000 nM in healthy individuals; ≈50 nM in CD and UC patients (91, 93)] and the fact that DRD3 may be selectively stimulated at low dopamine concentrations, these results suggest that low dopamine levels present in the inflamed gut mucosa favour the inflammatory potential of CD4^+^ T-cells, thus promoting chronic inflammation. Accordingly, DRD3-deficiency in CD4^+^ T-cells results in a significant attenuation in disease manifestation in a mouse model of inflammatory colitis (102). Of note, equivalent to the situation of inflammatory colitis, we have shown that DRD3-deficiency in CD4^+^ T-cells results in a complete attenuation of MPTP-induced neurodegeneration and the treatment of wild-type mice with a selective DRD3-antagonist significantly reduces the development of PD in two different animal models (46, 103). Conversely, high dopamine concentrations in the gut of healthy individuals would stimulate DRD2, favouring the production of the anti-inflammatory cytokine IL-10 by CD4^+^ T-cells (104) and suppressing both increased motility and ulcer development (105). Indeed, a genetic polymorphism of DRD2 gene, which results in decreased receptor expression, has been reported as a risk factor for IBD (106). In this regard, although the frequency of Tregs was not changed in the gut, suppressor function of intestinal Tregs was compromised in inflammatory colitis (107), a condition associated to decreased dopamine levels (92). Interestingly, the impairment of suppressive Tregs function was abolished by the administration of cabergoline, a DRD2 agonist (107). Taken together these findings suggest that, whereas DRD2-signalling in CD4^+^ T-cells would promote suppressive activity and tolerance in a healthy gut mucosa containing high dopamine levels, the selective DRD3-signalling in CD4^+^ T-cells promotes the inflammatory potential of T-cell mediated immunity in the inflamed gut mucosa containing low dopamine levels.

Beside the involvement of dopaminergic dysregulation in gut inflammation, arises the question of why dopamine levels are reduced in these conditions. In this regard, different non-exclusive mechanisms might be involved, including changes in the composition of gut microbiota (dysbiosis; see section Gut Microbiota as a Master Regulator of T-cell Mediated Immunity), the loss of catecholaminergic neurons of the enteric nervous system (see section Introduction), and the limited synthesis and secretion of dopamine as a consequence of α-synuclein aggregation in the neurons of the enteric nervous system. According to the latter mechanism involved in the reduction of dopamine levels, it has been described that healthy α-synuclein plays a role in the transport of presynaptic vesicles to nerve terminals. However, the aggregation of this protein, results in impaired secretion of presynaptic vesicles (99). Thus, similar to the reduction of dopamine levels observed in the striatum of PD patients as a consequence of Lewy bodies formation in the dopaminergic neurons of the nigrostriatal pathway, dopaminergic neurons of the enteric nervous system might result in impaired secretion of dopamine upon Lewy bodies formation in the gut mucosa. Moreover, a recent study performed with endoscopic biopsies and blood samples obtained from children with documented gut inflammation show that α-synuclein expression in enteric neurites correlated with the degree of the gut wall inflammation and that both monomeric or oligomeric forms of this protein induced dendritic cells maturation and triggered the recruitment of CD11b^+^ neutrophils and monocytes, suggesting a role of α-synuclein in the activation of innate immunity in the gastrointestinal tract (69). Taken together, these findings suggest that not only microbiota dysbiosis and the loss of neurons of the enteric nervous system would be involved in the reduction of dopamine levels associated to gut inflammation, but also the aggregation of α-synuclein should play a relevant contribution to this issue. Moreover, α-synuclein seems to play a direct role stimulating innate immunity in the gut mucosa. Thus, it seems that different mechanisms affecting gut homeostasis converge in the upregulation of α-synuclein and the reduction of dopamine levels in the gut mucosa, which plays a key role as a danger-signal stimulating high-affinity dopamine receptors expressed in T-cells, promoting inflammation.

## Short-Chain Fatty Acids as Regulators of T-cell Mediated Immunity

A major class of mediators produced by gut microbiota corresponds to the SCFAs derived from bacterial fermentation products, including acetate, propionate, and butyrate, among others. These mediators might act on T-cell physiology either by stimulating G-protein coupled receptors or by modifying the activity of epigenetic enzymes that regulate gene transcription (108). Regarding the SCFAs effects mediated by G-protein coupled receptors, a number of studies have shown GPR41, GPR43, and GPR109A as the main SCFAs receptors present in T-cells. For instance, the stimulation of GPR41 by propionate, and with lower affinity by butyrate, has been shown to attenuate Th2 responses. Thereby GPR41 stimulation exerts a protective effect in allergic inflammation in the airways (109). In addition, the stimulation of GPR43, which recognizes acetate and propionate with similar affinities, has been described to exert a potent immunosuppressive effect attenuating gut inflammation. In this regard, it has been shown that GPR43 expression is favoured in colonic Tregs and its stimulation induces the expansion and promotes the suppressive activity of these cells (30). Similarly, the GPR109A, which is stimulated by butyrate and niacin with similar affinity, induces anti-inflammatory features in colonic macrophages and dendritic cells, thus favouring the expansion and suppressive activity of Tregs and concomitantly attenuates the pro-inflammatory potential of Th17 cells (110). Importantly, one of the mechanisms by which SCFAs shape T-cell behaviour is based on the ability of these mediators to inhibit histone deacetylase and thus modifying the epigenetic landscape of T-cells chromatin (111). In this regard, it has been shown that butyrate and propionate increase the acetylation of the *foxp3* locus, favouring a higher expression of Foxp3 and consequently an enhanced Tregs differentiation and stronger suppressive activity (29). In the same direction, it has been described that SCFAs increase the mucosal barrier function in the duodenum by reducing epithelium permeability and increasing the secretion of bicarbonate to the lumen, thus avoiding the immune recognition of luminal bacteria and the consequent inflammation (112). Furthermore, a recent study has shown that Clostridium butyricum B1, by producing butyrate, favours the differentiation of CD4+ T-cells into Th22 (113), a subset of T-cells that produce IL-22, upregulating tight junctions expression in epithelial cells of the gut mucosa, thus increasing the barrier function (54). Taken together these studies indicate that SCFAs derived from intestinal commensal bacteria exert an anti-inflammatory effect in the mucosal immunity of the gut by both, promoting Tregs function and increasing barrier function.

As stated above, recent studies have consistently found a dysbiosis in the gut microbiota of PD patients as well as in a number of animal models of PD (10, 11, 114). In this regard, Unger and collaborators analysed the SCFAs contained in fecal samples obtained from 34 PD patients and 34 age-matched controls and found a significant reduction of SCFAs in PD patients (114). According to these results, reduced production of SCFAs has been related with dysfunctional Tregs activity and consequent gut inflammation (29, 110). Furthermore, it has been recently shown that germ-free mice present an altered organization of tight junctions in the BBB involving a down-regulation of occludin and claudin 5 and consequently an increased permeability of this barrier. Of note, the proper occludin and claudin 5 expression and reduced BBB permeability was restored by monocolonization with different SCFAs-producer bacterial strains (115). Moreover, another study has recently shown that GPR41 stimulation by propionate in the BBB attenuates the expression of the LPR-1 transporter, thus providing protection of the BBB from oxidative stress (116). In apparent controversy with the study performed by Unger and collaborators, as stated above (section Dopaminergic Regulation of T-cell Mediated Immunity) the study performed by Sampson and collaborators found that when microbiota was depleted from ASO mice, several parkinsonian manifestations were significantly reduced or even disappeared, a condition associated with reduced SCFAs production (10). However, when germ-free ASO mice were reconstituted with microbiota obtained from PD patients, they showed decreased levels of acetate but increased levels of propionate and butyrate in fecal samples, which was associated with stronger parkinsonian manifestations in comparison with animals reconstituted with microbiota obtained from healthy individuals (10). Thus, together these studies demonstrate that dysbiosis associated to PD involves an alteration in the production of SCFAs. The precise alterations in the level of SCFAs might subsequently trigger the loss of immune tolerance in the gut mucosa and the failure of BBB functions. Thereby, the dysbiosis involved in PD might be a key factor triggering T-cell autoimmunity directed to Lewy bodies-derived antigens.

## Involvement of gut-microbiota in Autoimmunity

Since PD physiopathology involves an adaptive immune response mediated by CD4^+^ T-cells, and these cells exert an inflammatory effect in response to self-antigens (i.e., lewy bodies), PD gathers the characteristics to be considered an autoimmunity (4). Accordingly, it has been shown that PD development requires both autoreactive CD4^+^ T-cells (37, 41, 49, 117) and also proper class II MHC able to present auto-antigens-derived peptides to autoreactive CD4^+^ T-cells (118). At this point it is important to note that different human leukocyte antigen (HLA) alleles involve different peptide-binding preferences and affinities and consequently activation of different T-cell clones and different quality of activation of pro-inflammatory or anti-inflammatory T-cells (119). Thereby a key genetic factor to take in consideration for autoimmune disorders is the HLA polymorphism. According to the autoimmune nature of PD, alleles HLA-DQB1^*^06 and HLA-DRB1^*^0301 have been shown to be significantly more frequent in PD patients (120, 121). Notably, the allele HLA-DRB1^*^0301 (121) has also been associated to the genetic susceptibility of some classical autoimmune diseases such as diabetes (122) and multiple sclerosis (MS) (123). In addition, a recent study analysed the binding of different alleles of class II MHC to peptides derived from autoantigens associated to PD and found a positive association between alleles HLA-DRB1^*^1501, HLA-DRB1^*^0304, and HLA-DRB5^*^0101 and the binding to autoantigens-derived peptides in PD patients (41). Together these findings indicate that, in addition to the classical polymorphism of components of the mitochondrial and autophagy machinery associated to PD risk (124), the HLA haplotype constitutes a key genetic factor associated to the susceptibility to develop PD.

As indicated in section Dopaminergic Regulation of T-cell Mediated Immunity, gut microbiota has been shown to play a key role in triggering PD development, however the underlying mechanism is still unclear. On the other hand, gut microbiota has been described to be also a fundamental factor in the development of other autoimmune disorders (34, 87, 88, 125). Thereby the understanding of the mechanisms involved in how microbiota trigger autoimmune responses in other disorders might give the clues to understand how microbiota may induce the development of PD in susceptible individuals. An interesting example is the study of how microbiota promotes the development of uveitis carried out by Horai and collaborators (34). In this study the authors used transgenic mice (R161H mice) bearing CD4^+^ T-cells expressing a TCR specific for the recognition of the interphotoreceptor retinoid binding protein (IRBP), a component of the retina. These animals spontaneously developed uveitis, an autoimmune response to the retina, which constitutes a major cause of blindness in humans. However, when the authors depleted microbiota by treatment with broad-spectrum antibiotics or by breeding these animals in germ-free conditions, the development of uveitis was strongly attenuated. Interestingly, the activation and acquisition of the Th17 inflammatory phenotype of transgenic CD4^+^ T-cells took place early in the small intestine, before the infiltration of these autoreactive T-cells into the eye. Strikingly, when authors developed R161H mice knockout for the cognate-antigen of transgenic T-cells (IRBP), these animals still displayed a vigorous activation and acquisition of the Th17 phenotype of transgenic CD4^+^ T-cells in the small intestine, a process that was dependent on the presence of SFB in the intestine. Thus, this study demonstrated that the activation of autoreactive T-cells is induced by non-cognated antigens present in the SFB or alternatively by antigens coming from SFB with molecular mimicry with retinal antigens. Importantly, this study constitutes an example illustrating how microbiota can trigger autoimmunity by molecular mimicry or by cross-reactivity between autoantigens and bacterial antigens present in the gut microbiota.

Due to its relevance in the development of autoreactive T-cell mediated responses, gut microbiota represents an important environmental factor able to trigger autoimmunity in susceptible individuals. An illustrative example for this, is a recent study performed with 34 monozygotic twin pairs discordant for MS. In this study Berer and collaborators show that when microbiota from the MS twin is transplanted into a transgenic mouse model of spontaneous brain autoimmunity, mice developed autoimmunity with higher incidence than when transplanted with microbiota coming from the healthy twin (125). Importantly, when mice were transplanted with microbiota obtained from MS twins, immune cells present in the gut mucosa produced significantly lower levels of the anti-inflammatory cytokine IL-10 than those immune cells of animals receiving microbiota coming from healthy twins. In addition, the analysis in the composition of gut microbiota shows clear differences between healthy and MS twins (125). Thus, this study represents an illustrative example of how the precise composition of gut microbiota might play an important role favouring the induction of an immunosuppressive environment in the gut mucosa and how changes in the microbiota composition may induce the loss of this environment triggering the development of inflammatory and autoimmune disorders.

Considering the previous example, the parallelism between MS and PD results intriguing: (i) both inflammatory disorders involve an autoimmune component mediated by CD4^+^ T-cells specific for central nervous system antigens, (ii) in both cases gut microbiota seems to play a key role triggering the activation of autoreactive T-cells in the gut mucosa, and (iii) the risk to develop both pathologies involve a genetic association with HLA-polymorphism. Since the autoimmune nature of MS and its relationship with gut-microbiota have been much longer explored than in PD, maybe we should take advantage from the knowledge about the mechanistic involvement of microbial consortium and adaptive immunity in the physiopathology of MS and from the successful immunotherapies developed in MS to better understand and to fight against PD. In this regard, several studies in MS and animal models (EAE) have shown that microbial organisms might trigger the activation of autoreactive T-cells specific for central nervous system antigens either through molecular mimicry or via bystander activation. Moreover, several gut microbiota-derived metabolites and bacterial products have been described to interact with the immune system to modulate central nervous system autoimmunity (126). In addition, the involvement of some accessory immune cells that results key players in the CD4^+^ T-cell mediated inflammation associated to MS should be considered in PD, such as γδT-cells, which strongly inhibit the suppressive activity of Tregs in the central nervous system (127), or peripheral macrophages infiltrating the brain, which results much more relevant than microglia promoting neuroinflammation in response to GM-CSF (128). Finally, it is important to mention that there is already some evidence showing significant therapeutic effects in animal models of PD, induced by MPTP, 6-OHDA or rotenone, when treated with therapies used in MS to block the infiltration of autoreactive CD4^+^ T-cells into the brain, such as Fingolimod/FTY720 (129–131). These findings encourage to further explore the mechanistic parallelism between both pathologies and also to evaluate the afficacy of other immunotherapies used in MS as a potential treatment for PD, such as rituximab (anti-CD20 monoclonal antibody geared to deplete B-cells) or natalizumab (monoclonal antibody mediating the blockade of α4-integrin, required by T-cells to infiltrate the brain).

## Conclusions

Several lines of evidence point to the hypothesis that PD development is triggered in the intestine, including the early loss of neurons of the enteric nervous system, mucosal inflammation, and the generation of α-synuclein inclusions in the gut. Importantly, PD is positively associated to IBD and both disorders involve a CD4^+^ T-cell driven chronic inflammation. Furthermore, the development of both PD and IBD has been found to be strongly dependent on the composition of gut microbiota. Notably, some components of gut microbiota might trigger the generation of α-synuclein inclusions in the gut, which constitutes the main source of autoantigens driving the CD4^+^ T-cell response in PD. Furthermore, gut microbiota produces several mediators in the gut mucosa, such as SCFAs, dopamine and other metabolites, which stimulate their receptors in T-cells, thus shaping the adaptive immune response. In addition, some gut microbiota strains have been shown to trigger autoimmunity by providing molecular mimicry or cross-reactivity with self-antigens. Thus, we propose here that CD4^+^ T-cell response to Lewy bodies-derived antigens is triggered initially by gut microbiota, inducing an early gut inflammation and later PD (Figure 1).

## Author Contributions

RP designed the study. JC-A, DE, and RP acquired data, analysed data, and wrote the manuscript.

### Conflict of Interest Statement

The authors declare that the research was conducted in the absence of any commercial or financial relationships that could be construed as a potential conflict of interest.

## Abbreviations

ASO: transgenic mice over-expressing human α-synuclein
α-syn: α-synuclein
APCs: antigen-presenting cells
BBB: blood-brain-barrier
CD: Crohn's disease
CD*n*: cluster of differentiation *n*
DRD*n*: dopamine receptor D*n*
Foxp3: forkhead box P3
GFAP: Glial Fibrillary Acidic Protein
GPR*n*: G-protein coupled receptor *n*
HLA: human leukocyte antigen
HSV1: herpes simplex virus 1
IBD: inflammatory bowel diseases
IFN-γ: interferon γ
IL-*n*: interleukin *n*
IRBP: interphotoreceptor retinoid binding protein
LPR-1: Low-density lipoprotein receptor-related protein 1
LPS: lipopolysaccharide
MHC: Major Histocompatibility Complex
MLN: mesenteric lymph nodes
MPTP: 1-methyl-4-phenyl-1,2,3,6-tetrahydropyridine
MS: Multiple Sclerosis
NF-κB: nuclear factor kappa-light-chain-enhancer of activated B cells
PD: Parkinson's disease
RAG1: recombination-activating-gen-1
RAG1KO: RAG1 knockout
RNS: reactive nitrogen species
ROS: reactive oxygen species
SCFAs: short-chain fatty acids
SCID: severe combined immunodeficiency
SFB: segmented filamentous bacteria
TCR: T-cell receptor
Th*n*: T helper *n*
TLRs: Toll like receptors
TLR*n*: Toll like receptor *n*
TNF-α: Tumor Necrosis Factor α
Tregs: regulatory T cells
UC: ulcerative colitis.
